# Peripheral injury of pelvic visceral sensory nerves alters GFRα (GDNF family receptor alpha) localization in sensory and autonomic pathways of the sacral spinal cord

**DOI:** 10.3389/fnana.2015.00043

**Published:** 2015-04-10

**Authors:** Shelley L. Forrest, Sophie C. Payne, Janet R. Keast, Peregrine B. Osborne

**Affiliations:** ^1^Pain Management Research Institute (Kolling Institute), University of Sydney at the Royal North Shore HospitalSydney, NSW, Australia; ^2^Department of Anatomy and Neuroscience, The University of MelbourneMelbourne, VIC, Australia

**Keywords:** nerve injury, sacral spinal cord, dorsal horn, visceral afferents, parasympathetic nervous system, preganglionic, GDNF family of ligands (GFL), pelvic pain

## Abstract

GDNF (glial cell line-derived neurotrophic factor), neurturin and artemin use their co-receptors (GFRα1, GFRα2 and GFRα3, respectively) and the tyrosine kinase Ret for downstream signaling. In rodent dorsal root ganglia (DRG) most of the unmyelinated and some myelinated sensory afferents express at least one GFRα. The adult function of these receptors is not completely elucidated but their activity after peripheral nerve injury can facilitate peripheral and central axonal regeneration, recovery of sensation, and sensory hypersensitivity that contributes to pain. Our previous immunohistochemical studies of spinal cord and sciatic nerve injuries in adult rodents have identified characteristic changes in GFRα1, GFRα2 or GFRα3 in central spinal cord axons of sensory neurons located in DRG. Here we extend and contrast this analysis by studying injuries of the pelvic and hypogastric nerves that contain the majority of sensory axons projecting to the pelvic viscera (e.g., bladder and lower bowel). At 7 d, we detected some effects of pelvic but not hypogastric nerve transection on the ipsilateral spinal cord. In sacral (L6-S1) cord ipsilateral to nerve injury, GFRα1-immunoreactivity (IR) was increased in medial dorsal horn and CGRP-IR was decreased in lateral dorsal horn. Pelvic nerve injury also upregulated GFRα1- and GFRα3-IR terminals and GFRα1-IR neuronal cell bodies in the sacral parasympathetic nucleus that provides the spinal parasympathetic preganglionic output to the pelvic nerve. This evidence suggests peripheral axotomy has different effects on somatic and visceral sensory input to the spinal cord, and identifies sensory-autonomic interactions as a possible site of post-injury regulation.

## Introduction

Peripheral sensory nerves are easily damaged by common surgeries, accidental trauma and disease, which can in turn cause persistent post-surgical and neuropathic pain that is difficult to treat (Costigan et al., 2009; Jensen et al., 2011; Schug, 2012). A large epidemiological study of persistent post-surgical pain has identified the location of surgical neural damage as a risk factor (Johansen et al., 2012; Schug, 2012)—as prevalence is high following surgery on the back, neck, hip and limbs, but is low after surgeries on the abdomen and pelvic viscera. This clinical finding identifies a significant limitation in preclinical studies of the relationship between nerve injury and pain, as most reports use nerve injury models where only somatic (cutaneous, muscle and joint) afferents are damaged (von Hehn et al., 2012). By far the most extensively studied models are those involving injuries to the sciatic nerve projecting to the hindlimbs or cranial nerves projecting in the neck and head. As a result, there is relatively little comparative information available on the outcome of similar injuries to major nerves that are mostly comprised of visceral sensory afferents.

Peripheral nerve injuries have complex effects on rodent primary sensory neurons (Christie and Zochodne, 2013; Scheib and Höke, 2013). It is well established that injuring peripheral axons initiates a pro-regenerative state that supports axon growth, reinnervation of target organs and recovery from sensory deficits. However, other forms of neuroplasticity expressed by primary sensory neurons after nerve injury are pathophysiological and result in persistent sensory dysfunction and pain (von Hehn et al., 2012). The effects of nerve injury are not restricted to the peripheral axons of primary sensory neurons, as central axonal projections in the spinal cord dorsal horn can also show evidence of chemical and structural neuroplasticity (Navarro et al., 2007; Yang et al., 2014). For example, sciatic nerve injury causes remodeling of the axons of peptidergic and non-peptidergic primary afferent C-fiber axons containing calcitonin gene-related peptide (CGRP) and isolectin-B4 respectively (Bailey and Ribeiro-da-Silva, 2006; Casals-Díaz et al., 2009; Keast et al., 2010). However, again these changes have mostly been characterized in the L4-L5 segments of the lumbar spinal cord using somatic nerve injury models involving different branches of the sciatic nerve.

To investigate the impact of visceral nerve injury on the spinal cord this study has transected pelvic and hypogastric nerves, which in rat are visceral nerves essential for the micturition reflex and other urogenital functions (Yoshimura, 1999; Keast, 2006). There are dramatic species differences in the organization of the sensory and autonomic components of these projection pathways but in humans the homologous nerves associated with the pelvic (inferior hypogastric) plexus are often damaged by routine surgical procedures, resulting in bladder, bowel and sexual dysfunction (Walsh and Donker, 1982; Maas et al., 2003). The pelvic nerve in rat is a mixed nerve comprising sensory Aδ- and C-fiber axons that input to L6-S1 spinal cord segments, parasympathetic preganglionic axons that project from L6-S1 spinal cord and function in sacral autonomic regulation, and sympathetic postganglionic axons from paravertebral ganglia (Keast, 2006). The hypogastric nerve in rat extends from the inferior mesenteric to the major pelvic ganglia. It is also a mixed nerve comprising sensory Aδ- and C-fiber axons that project to upper lumbar (L1/L2) spinal cord segments in rat; spinal sympathetic preganglionic axons projecting from the L1-L2 segments; as well as some sympathetic postganglionic axons projecting from the inferior mesenteric ganglion.

Very few studies have examined the impact of injury on pelvic visceral sensory neurons in any species. An early study in cats found that transecting the pelvic nerve decreased C-fibers expressing vasoactive intestinal polypeptide in the dorsal horn, but in contrast to somatic nerve injury (transection of sciatic and pudendal nerves), the peptidergic C-fibers expressing galanin and somatostatin were not affected (Anand et al., 1991). We are not aware of any equivalent studies in rodents, but our group has identified changes in these nerves after an inflammatory challenge (Forrest and Keast, 2008). Using a model of cyclophosphamide-induced bladder cystitis, we found that non-peptidergic afferents in sacral dorsal horn upregulate glial cell line-derived neurotrophic factor (GDNF) receptor alpha1 (GFRα1), which is the ligand-binding receptor of GDNF (Airaksinen and Saarma, 2002). We do not know if this was due to a direct effect of the inflammatory environment or the acute structural damage of sensory terminals in the bladder that occurs in this model. However, we have also determined that GFRα receptors are affected by injuring peripheral sensory axons in a rat somatic nerve injury model (Keast et al., 2010). We found that sciatic nerve injury increases GFRα1 and the artemin receptor, GFRα3, in sacral spinal cord and down-regulates the neurturin receptor, GFRα2 in lumbar cord. GFRα1 and GFRα2 are expressed in separate populations of non-peptidergic C-fiber afferents, GFRα1 is also expressed in some myelinated afferents, and GFRα3 is exclusively expressed in a sub-population of peptidergic C-fibers (Orozco et al., 2001; Kalous et al., 2007, 2009; Ernsberger, 2008; Keast et al., 2010). On this basis, our aim here was to determine if axotomy of peripheral visceral axons in the pelvic or hypogastric nerves in rat have similar or different effects on the spinal cord distribution of four distinct populations of afferent fibers identified with GFRα1, GFRα2, GFRα3 and CGRP (which includes the GFRα3-positive population). We also investigated if these injuries had any effect on GFRα expression in neuronal somata of the sacral parasympathetic nucleus (SPN), an area containing preganglionic neurons would be axotomised by pelvic or hypogastric nerve transection.

## Methods

### Animals and Surgical Procedures

All procedures complied with the Australian Code for the Care and Use of Animals for Scientific Purposes (National Health and Medical Research Council of Australia) and were approved by Animal Ethics Committees at the University of Sydney or the University of Melbourne. Adult male Sprague-Dawley rats (20 in total, aged 6–8 weeks; Animal Resources Centre, Murdoch, WA) were housed in groups of 3, under a 12 h light-dark cycle, with *ad libitum* access to water and standard chow.

All surgical procedures were performed under isoflurane anesthesia (3% in oxygen for induction, 1.5–2% for maintenance). Four types of nerve injury surgeries (unilateral transection of the pelvic (*n* = 11) or hypogastric (*n* = 4) nerves; bilateral transection of both the pelvic and hypogastric (*n* = 3) nerves or pelvic nerve only (*n* = 2)) were performed using published methods (Kalous and Keast, 2010; Peddie and Keast, 2011). In brief, the lower abdominal cavity was opened via a midline excision to expose the pelvic organs, which were displaced to access the pelvic ganglia located on the dorsolateral aspect of the prostate gland. The hypogastric and/or pelvic nerves were then isolated from underlying prostate tissue with fine forceps and cut with iris scissors at approximately 1 mm from the ganglion, after which the abdominal muscle and skin were sutured. All rats were closely monitored following surgery. As bilateral transection of the pelvic nerve prevents micturition, animals that received this surgery had their bladder emptied manually at intervals of less than 12 h.

### Tissue Preparation

Seven days after surgery, rats were deeply anesthetized with sodium pentobarbitone (80 mg/kg i.p.) and transcardially perfused with 0.9% saline containing 1.25% sodium nitrite and 0.036% heparin, followed by freshly made 4% paraformaldehyde fixative in 0.1 M phosphate buffer (PB, pH 7.4). Spinal cords were removed and post-fixed overnight in the same fixative at 4°C, then washed in 0.1 M phosphate buffered saline (PBS, pH 7.2) and stored at 4°C in PBS containing 0.1% azide. Fixed spinal cords were marked by a superficial cut to the ventrolateral gray matter to identify the side ipsilateral to nerve injury and segmented into sacral (L6-S1) and upper lumbar (L1–2) regions. They were then cryoprotected overnight in PBS containing 30% sucrose and cut on a cryostat. Transverse sections (40 μm) were collected in a 1 in 4 series so that sections processed for the same substance were sampled at least 160 μm apart. To visualize the rostrocaudal extent of the SPN, horizontal sections (40 μm) of the L4-S2 segment were also collected in a 1 in 2 series so that sections processed for the same substance were sampled at least 80 μm apart.

### Chromagen Immunohistochemistry

All sections were processed free-floating using a glucose oxidase/nickel enhanced diaminobenzidine (DAB) method (Hamlin et al., 2007; Kalous et al., 2007, 2009). In brief, sections were washed in PB prior to incubations (30 min) in 50% ethanol, 50% ethanol containing 3% H_2_O_2_ to block endogenous peroxidase activity, and a blocking solution of 5% normal horse serum (NHS) in PB. Transverse sections were then incubated on a shaker (48 h at room temperature) in affinity purified antisera raised against goat GFRα1 (1:400; R&D Systems, Minneapolis, MN, cat. no. AF560; RRID:AB_2110307), GFRα2 (1:1000, R&D Systems, cat. no. AF429; RRID:AB_2294621), GFRα3 (1:300, R&D Systems, cat. no. AF2645; RRID:AB_2110295) or rabbit CGRP (1:2000, Millipore, Vic, Australia, cat. no. RRID:AB_2068655). Details of antibody characterization are provided in previous reports (Kalous et al., 2007, 2009; Keast et al., 2010; Forrest et al., 2013, 2014) and additional references linked to their research resource identifiers.^1^ Horizontal sections were incubated in GFRα1 or GFRα3 antisera only. After washes in PB, sections were then incubated overnight in biotin-conjugated anti-goat or anti-rabbit affinity purified antisera (1:1000, Jackson ImmunoResearch Laboratories, West Grove, PA) raised in donkey. All antisera dilutions were made using PB containing 2% NHS and 0.2% triton X-100.

Following washes in PB, sections were then incubated (2 h at room temperature) in avidin-biotin complex (6 μl/ml, Vectastain Elite kit; Vector Laboratories, Burlingame, CA), followed by a 15 min incubation in a nickel DAB solution (2% nickel sulfate, 0.2% D-glucose, 0.04% ammonium chloride, and 0.025% DAB in 0.1 M sodium acetate buffer pH 6.0). Glucose oxidase (0.02%) was then added to obtain black chromagen staining, which was monitored using a dissecting microscope and stopped by washing in large volumes of acetate buffer. Sections were mounted from 0.9% saline onto slides (0.1% gelatinized), dried overnight, and dehydrated in ascending concentrations of ethanol prior to being cleared in histolene, and cover slipped with DPX water-free mounting media (Crown Scientific, Mulgrave, VIC, Australia).

### Image Analysis and Optical Density Measurements

**Figure 1:**
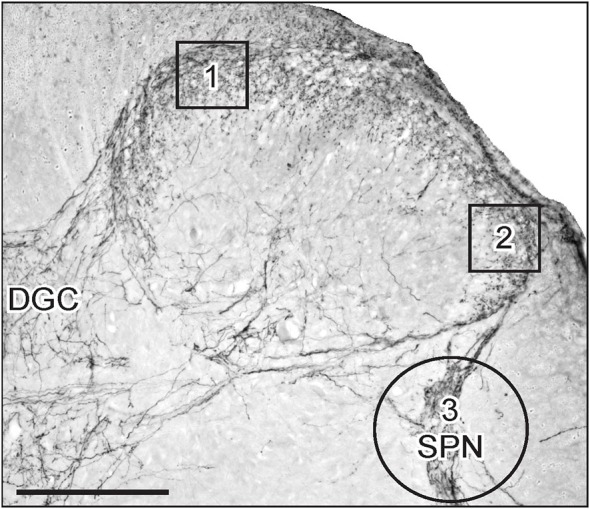
**Areas of interest (AOIs) used for densitometric image analysis following visceral nerve transection**. Image shows a micrograph of CGRP-immunoreactivity (IR) in the superficial dorsal horn and sacral parasympathetic nucleus (SPN) within sacral spinal cord. AOIs 1 and 2 (130 × 130 μm) were aligned with the medial and lateral margins of the superficial dorsal horn (boxes 1 and 2, respectively). Both AOIs were assessed for GFRα1, GFRα2, GFRα3 and CGRP in the lumbar and sacral spinal cord. The AOI encompassing the SPN (circle 3, diameter 250 μm) was assessed for GFRα1, GFRα3 and CGRP. Image also shows location of the dorsal gray commissure (DGC). Scale bar represents 200 μm.

The effect of unilateral pelvic or hypogastric nerve transection on optical density of immunohistochemical chromagen staining within the dorsal horn and SPN was measured by image analysis (Kalous et al., 2007, 2009; Keast et al., 2010). Figure 1 shows the three areas of interest (AOIs) used for this analysis, which were in accordance with our previous study of the effect of experimental cyclophosphamide-induced cystitis on GFRα-immunoreactivity (IR) in sacral spinal cord (Forrest and Keast, 2008). Images were captured under a 10× objective with an Olympus BX51 microscope (Olympus Australia, Melbourne, Vic) and a Zeiss AxioCam HRc camera controlled by Axiovision 4.2 software (Carl Zeiss, Australia), and saved as 8-bit monochrome (1300 × 1030 pixels) TIFF files. The microscope illumination and camera acquisition settings were the same for all images, and were set to maximize the dynamic range with no saturated pixels (Kalous et al., 2007, 2009; Keast et al., 2010).

The AOIs in the medial and lateral dorsal horn and SPN on the ipsi- and contralateral side of the same section. For quantitative analysis of immunostaining intensity, images ipsi- and contralateral to injury were taken from six randomly selected lumbar and sacral sections. Optical density was assessed in areas of interest (AOIs, 130 × 130 μm; Figure 1, boxes 1 and 2) in the dorsal horn and in the SPN (circular AOI, diameter = 250 μm; Figure 1, circle 3), demonstrated in Figure 1. AOI boxes 1 and 2 were used to assess immunostaining intensity of each antibody in the dorsal horn, but AOI circle 3 was used to assess immunostaining intensity for GFRα1, GFRα3 and CGRP in the SPN. The circular AOI was large enough to include neurons and fibers within the SPN and afferent fibers that terminated dorsal to the SPN. The optical density value of an antibody was obtained from all six sections and averaged to obtain a single value for each AOI per animal. For each antibody, the mean optical density value of the white matter was subtracted. The resultant changes in optical density could reflect changes in the staining intensity of fibers or fiber density within the AOI. Changes in optical density were equated to changes in staining intensity of fibers or changes in fiber density within the AOI.

### Neuronal Counts

GFRα1-IR neurons were counted in the SPN on the ipsi- and contralateral sides after unilateral transection, and on one side after bilateral transection. GFRα1-positive neurons were counted in 5 horizontal spinal cord sections and data expressed as the mean ± SEM of the total number of neurons per section with no stereological correction.

### Statistics and Figure Production

All data are expressed as the mean ± SEM and *P* < 0.05 were regarded as statistically significant. Statistical analyses were performed using SPSS v16 for Mac (Chicago, IL) or GraphPad Prism 5.0a (GraphPad Software, La Jolla, CA). Differences between sides of GFRα1–3 and CGRP immunostaining in the medial and lateral dorsal horn and SPN were analyzed using paired *t*-tests. For figure production, minor adjustments were made to brightness and contrast to best represent the immunostaining as viewed directly under the microscope (Adobe InDesign and Photoshop CS2; Adobe Systems, San Jose, CA, USA).

## Results

We examined the patterns of neuronal IR in regions of the rat sacral (L6/S1)(Figure 1) and upper lumbar (L1-L2) spinal cord that receive sensory input from the pelvic and hypogastric nerves, respectively. Transganglionic tracing studies in rat (Morgan et al., 1986; Nadelhaft and McKenna, 1987) show the sensory input at both levels distributes in Lissauer’s tract, superficial and deep dorsal horn, medial (MCP) and lateral collateral pathways (LCP), lamina X and the dorsal gray commissure (DGC; Figure 1). In sacral cord, there are also terminal arborizations in the sacral parasympathetic nucleus (SPN; Figure 1), which contains the parasympathetic preganglionic autonomic neurons that project in the pelvic nerve (Hancock and Peveto, 1979; Morgan et al., 1981; Nadelhaft and Booth, 1984).

**Figure 2:**
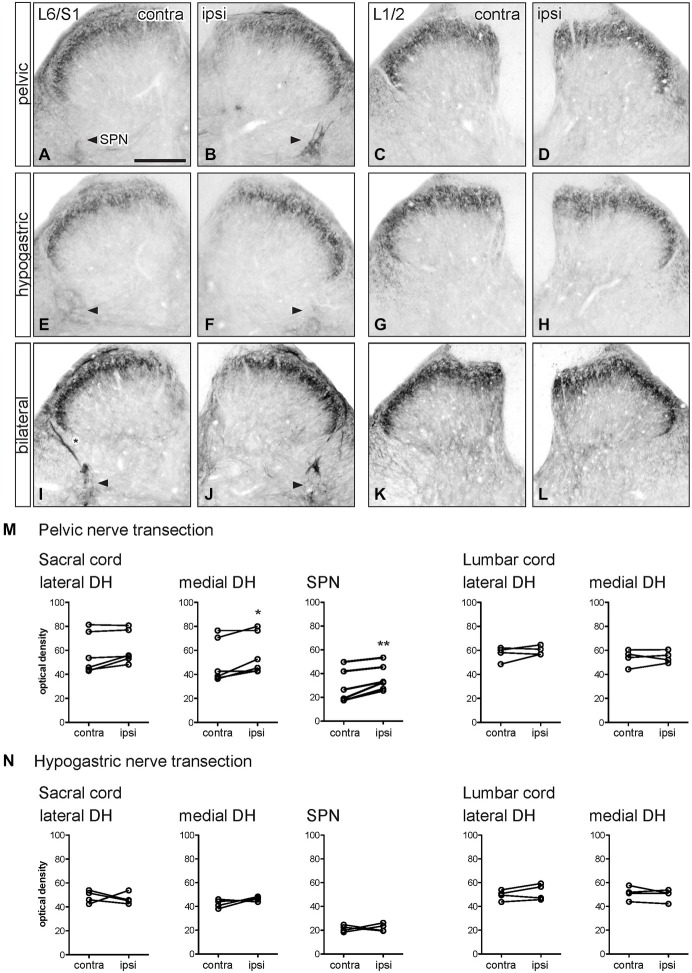
**Distribution of GFRα1-IR in sacral and lumbar spinal cord following visceral nerve transection**. For each type of injury, images were taken from left and right sides of the same section, and transverse segments of each spinal level from the same animal. **(A–L)** Images show GFRα1-IR in sacral (L6/S1) and lumbar (L1/2) spinal cord at 7 days after unilateral pelvic nerve transection **(A–D)**, unilateral hypogastric nerve transection **(E–H)** or bilateral pelvic and hypogastric nerve transection **(I–L). (M)** Quantification of GFRα1 optical density following unilateral pelvic nerve transection shows that in sacral cord there was no effect of injury in lateral dorsal horn, whereas a small but significant increase of GFRα1-IR was observed in the medial dorsal horn and sacral parasympathetic nucleus (SPN) ipsilateral to injury. In lumbar spinal cord, no effect of injury on GFRα1-IR was detected. **(N)** Hypogastric nerve transection had no effect on GFRα1-IR in sacral or lumbar spinal cord. Data represents the mean ± SEM (*n* = 6 rats for sacral data following unilateral pelvic nerve transection, *n* = 4 rats for lumbar data following unilateral pelvic and hypogastric nerve injury) and was analyzed using a paired *t*-test. The SPN in sacral cord is indicated with arrowheads **(A,B,E,F,I,J)**. Scale bar in A applies to all images and represents 200 μm.

To determine if unilateral lesions of the pelvic or hypogastric nerves affect the central axons of dorsal root ganglia (DRG) neurons we used digital image analysis to compare areas of interest (AOIs) in the spinal cord contralateral and ipsilateral to the injury. GFRα1- and GFRα2-IR were used as markers for the terminals of non-peptidergic C-fiber afferents (Forrest and Keast, 2008) whereas CGRP- and GFRα3-IR were used as markers for the terminals of peptidergic C-fiber afferents (Forrest and Keast, 2008; Keast et al., 2010). The distribution of all markers in the dorsal horn of the sacral (L6/S1) and lumbar (L1/2) spinal cord (Figures 2, 4–6) contralateral to both injuries was broadly consistent with previous reports (Forrest and Keast, 2008; Keast et al., 2010). However, as the experimental design was limited to a within-subject comparison of the contralateral and ipsilateral spinal cord, this precluded using quantitative analysis to detect an effect of nerve injury on the contralateral dorsal horn. Mean optical density measurements from three AOIs in medial and lateral superficial dorsal horn and SPN are summarized in Table 1 for each type of IR, spinal cord region and nerve injury group studied in the experiment.

**Figure 3:**
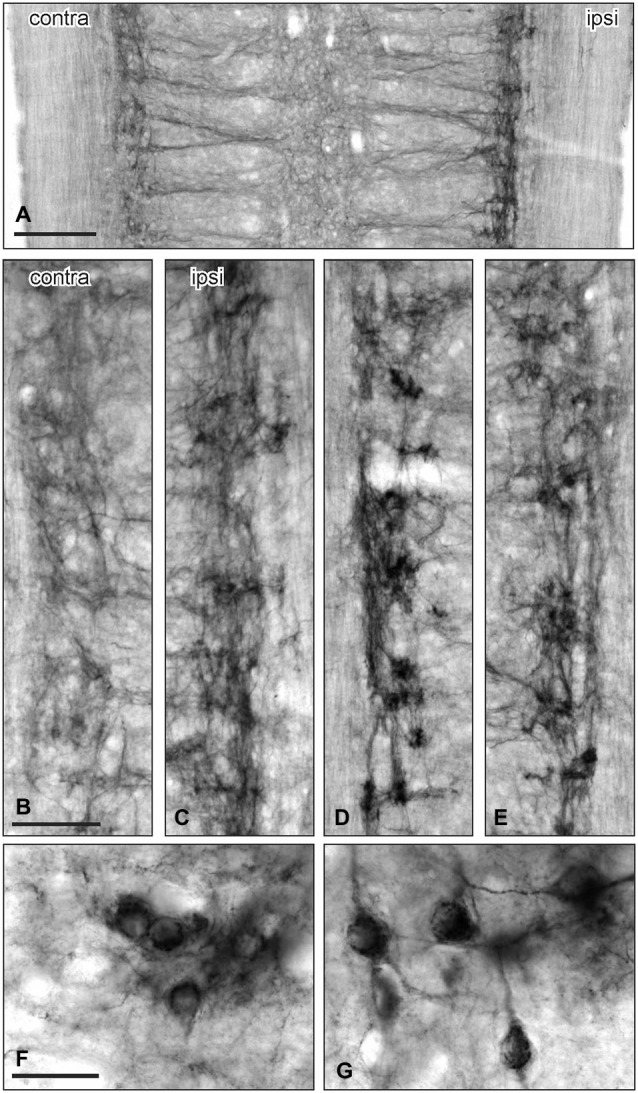
**GFRα1-IR in the sacral parasympathetic nucleus (SPN) viewed in horizontal spinal cord sections after pelvic nerve injury. (A–C)**: Asymmetric upregulation of GFRα1-IR in the SPN ipsilateral to a unilateral pelvic nerve transection. **(D,E)** Symmetric upregulation of GFRα1-IR in the SPN on both sides after bilateral pelvic nerve injury. **(F,G)** Examples of GFRα1-IR somata in SPN ipsilateral to pelvic nerve transection. Scale bars represent 200 μm **(A)**, 50 μm **(B–E)**, and 50 μm **(F,G)**.

**Figure 4:**
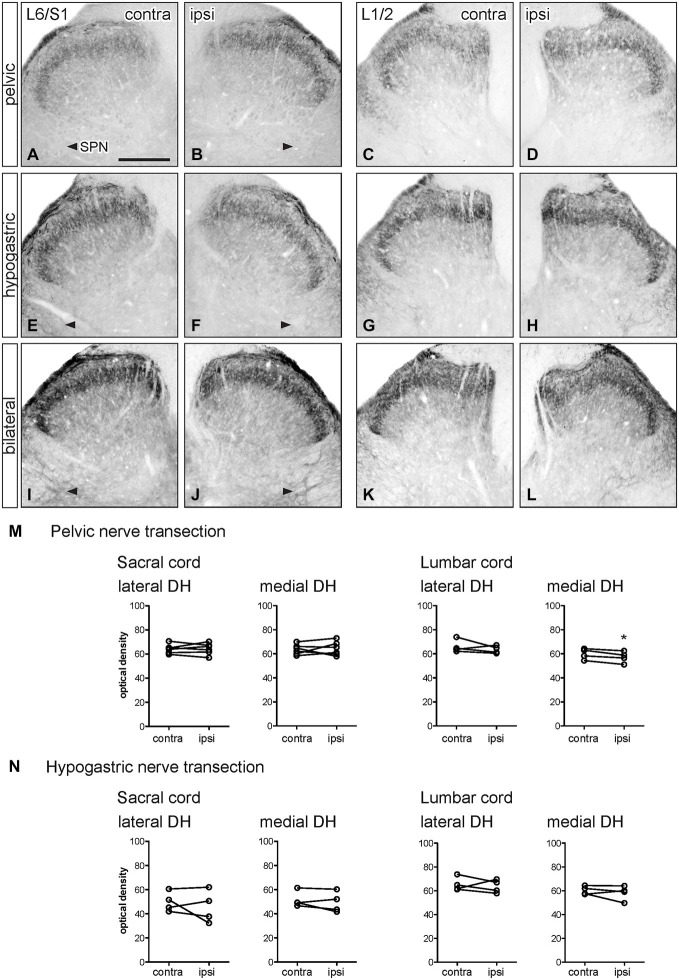
**Distribution of GFRα2-IR in sacral and lumbar spinal cord following visceral nerve transection**. For each type of injury, images were taken from left and right sides of the same section, and transverse segments of each spinal level from the same animal. **(A–L)** Images show GFRα2-IR in sacral (L6/S1) and lumbar (L1/2) dorsal horn at 7 days after unilateral pelvic nerve transection **(A–D)**, unilateral hypogastric nerve transection **(E–H)** or bilateral pelvic and hypogastric nerve transection **(I–L). (M)** Following unilateral pelvic nerve transection, no change in GFRα2-IR was seen in sacral dorsal horn. In lumbar spinal cord, there was a small but significant decrease in GFRα2-IR only in the medial dorsal horn. **(N)** Unilateral hypogastric nerve transection had no effect on GFRα2-IR in sacral or lumbar dorsal horn. Data represents the mean ± SEM (*n* = 6 rats for sacral data following unilateral pelvic nerve transection, *n* = 4 rats for lumbar data following unilateral pelvic and hypogastric nerve transection) and was analyzed using a paired *t*-test. Scale bar represents 200 μm.

**Figure 5:**
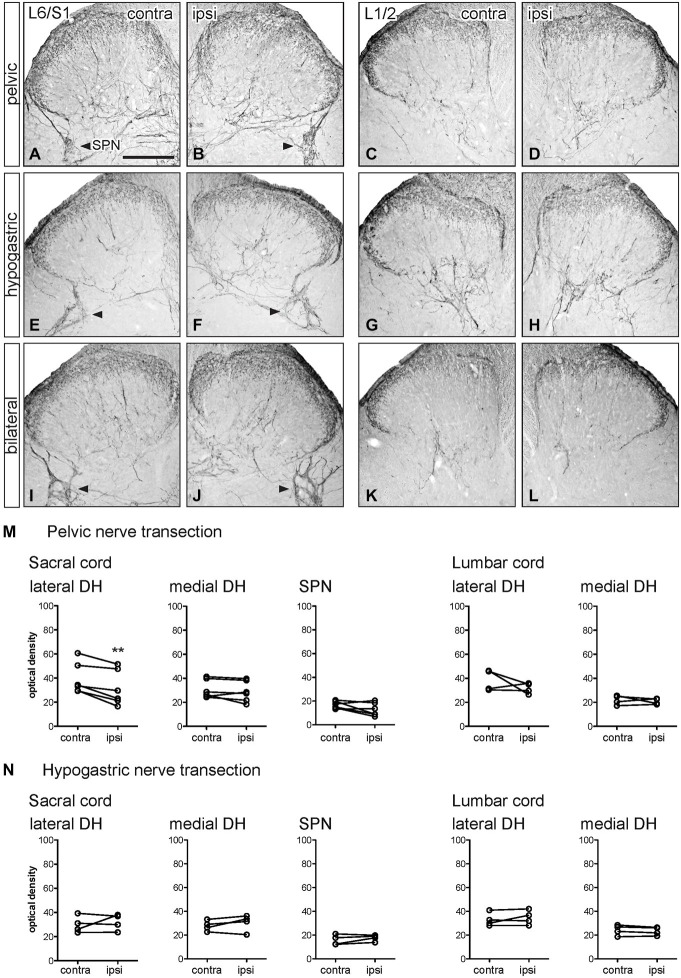
**Distribution of CGRP-IR in sacral and lumbar spinal cord following visceral nerve transection**. For each type of injury, images were taken from left and right sides of the same section, and transverse segments of each spinal level from the same animal. **(A–L)** Images show CGRP-IR in sacral (L6/S1) and lumbar (L1/2) dorsal horn 7 days after unilateral pelvic nerve transection **(A–D)**, unilateral hypogastric nerve transection **(E–H)** or bilateral pelvic and hypogastric nerve transection **(I–L). (M)** Following unilateral pelvic nerve transection, optical density analysis in sacral cord showed CGRP-IR decreased in only in the lateral dorsal horn. In lumbar cord, there was no change in CGRP-IR. **(N)** Hypogastric nerve transection had no effect on CGRP-IR in sacral or lumbar spinal cord. Data represents the mean ± SEM (*n* = 6 rats for sacral data following unilateral pelvic nerve transection, *n* = 4 rats for lumbar data following unilateral pelvic hypogastric nerve transection) and was analyzed using a paired *t*-test. SPN (L6/S1) is indicated with arrowheads **(A,B,E,F,I,J)**. Scale bar represents 200 μm.

**Figure 6:**
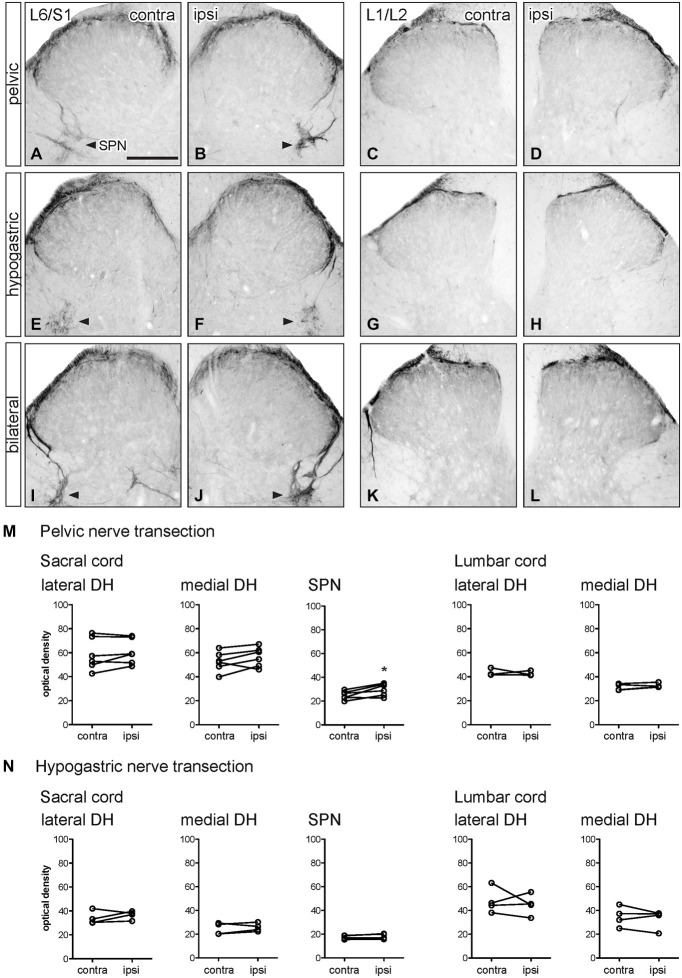
**Distribution of GFRα3-IR in sacral and lumbar spinal cord following visceral nerve transection**. For each type of injury, images were taken from left and right sides of the same section, and transverse segments of each spinal level from the same animal. **(A–L)** Images show GFRα3-IR in transverse sections of sacral (L6/S1) and lumbar (L1/2) dorsal horn at 7 days after unilateral pelvic nerve transection **(A–D)**, unilateral hypogastric nerve transection **(E–H)** or bilateral pelvic and hypogastric nerve transection **(I–L). (M)** Following pelvic nerve transection, optical density analysis in sacral cord showed no changes in GFRα3-IR in sacral dorsal horn, while an increase in GFRα3-IR was seen in the sacral parasympathetic nucleus (SPN). In lumbar spinal cord, there was no effect of injury on GFRα3-IR. **(N)** Hypogastric nerve transection had no effect on GFRα3-IR in sacral or lumbar spinal cord. Data represents the mean ± SEM (*n* = 6 rats for sacral data following unilateral pelvic nerve transection, *n* = 4 rats for lumbar data following unilateral pelvic hypogastric nerve transection) and was analyzed using a paired *t*-test. The SPN in sacral cord is indicated with arrowheads **(A,B,E,F,I,J)**. Scale bar represents 200 μm.

**Table 1:** **Optical density (mean ± SEM) of GDNF-family receptor alpha (GFRα)-immunoreactivity in sacral (L6/S1) and lumbar (L1/2) spinal cord segments following unilateral visceral nerve transection^1^**.

			Pelvic nerve transection			Hypogastric nerve transection		
Immunoreactivity (spinal level)		Location of area of interest	Contralateral	Ipsilateral	*p*-value^2^	Contralateral	Ipsilateral	*p*-value^2,4^
GFRα1	Sacral	Lateral dorsal horn	57.2 ± 6.9	61.7 ± 5.5	0.07^3^	48.5 ± 2.6	46.8 ± 2.5	0.73
		Medial dorsal horn	50.3 ± 7.5	56.7 ± 6.9	**0.04**	42.3 ± 1.8	46.3 ± 0.9	0.23
		SPN	28.7 ± 5.7	36.2 ± 4.5	**0.01**	21.3 ± 1.4	22.2 ± 1.7	0.77
	Lumbar	Lateral dorsal horn	57.3 ± 3.0	59.7 ± 1.9	0.36^4^	49.5 ± 2.1	52.2 ± 3.4	0.25
		Medial dorsal horn	53.9 ± 3.5	54.6 ± 2.4	0.75	51.1 ± 2.8	49.5 ± 2.5	0.45
GFRα2	Sacral	Lateral dorsal horn	64.3 ± 1.6	64.3 ± 1.9	0.97^3^	49.8 ± 4.1	45.7 ± 6.7	0.50
		Medial dorsal horn	63.6 ± 1.7	64.3 ± 2.4	0.74	51.6 ± 3.3	49.4 ± 4.3	0.38
	Lumbar	Lateral dorsal horn	66.1 ± 2.7	63.5 ± 1.6	0.38^4^	65.3 ± 2.9	63.7 ± 2.8	0.64
		Medial dorsal horn	59.9 ± 2.3	57.3 ± 2.4	**0.02**	60.3 ± 1.8	58.3 ± 3.1	0.45
CGRP	Sacral	Lateral dorsal horn	39.7 ± 5.3	31.6 ± 5.9	**0.01**^3^	29.9 ± 3.5	32.1 ± 3.6	0.56
		Medial dorsal dorn	30.9 ± 3.1	29.0 ± 3.5	0.26	27.8 ± 2.2	30.4 ± 3.5	0.28
		SPN	16.8 ± 1.3	12.9 ± 2.3	0.12	15.9 ± 2.1	17.7 ± 1.3	0.28
	Lumbar	Lateral dorsal horn	38.5 ± 4.4	31.8 ± 2.2	0.30^4^	32.9 ± 2.8	34.7 ± 3.0	0.38
		Medial dorsal horn	22.0 ± 1.9	20.7 ± 1.2	0.58	24.3 ± 2.2	23.4 ± 1.7	0.21
GFRα3	Sacral	Lateral dorsal horn	58.7 ± 5.5	60.9 ± 4.3	0.29^3^	33.9 ± 2.8	36.6 ± 1.8	0.37
		Medial dorsal horn	52.6 ± 3.4	56.6 ± 3.3	0.13	24.6 ± 2.5	25.7 ± 1.7	0.47
		SPN	24.9 ± 1.4	29.8 ± 2.1	**0.02**	17.5 ± 0.8	18.3 ± 1.2	0.12
	Lumbar	Lateral dorsal horn	43.3 ± 1.4	42.5 ± 0.9	0.69^4^	47.9 ± 5.4	44.9 ± 4.5	0.63
		Medial dorsal horn	31.4 ± 1.4	32.6 ± 0.9	0.27	34.8 ± 4.2	32.9 ± 4.1	0.49

*^1^For each rat a single estimate of the optical density expressed in arbitrary units was obtained by averaging measurements from six sections per rat*.

*^2^Ipsilateral vs. contralateral (Paired *t*-test). ^3^*n* = 6. ^4^*n* = 4. Abbreviation: SPN, sacral parasympathetic nucleus*.

### GFRα1 Immunoreactivity

GFRα1-IR terminals in the spinal cord contralateral to nerve injury (sacral: Figures 2A,E lumbar: Figures 2C,G) were distributed primarily in lamina II (outer) of the dorsal horn as previously described (Forrest and Keast, 2008; Kalous et al., 2009). Occasional weakly stained fibers also projected in the LCP and DGC, an area that contains autonomic preganglionic axons and interneurons (Morgan et al., 1986). In sacral cord, weakly stained terminal arborizations and some neuronal cell bodies were also present in the SPN. GFRα1-IR motor neurons were also found in the ventral horn (not shown).

Optical density measurements of GFRα1-IR in the sacral spinal cord 7 days after unilateral pelvic nerve transection (Table 1; Figures 2A,B,M) detected a significant increase in the medial superficial dorsal horn whereas in the lateral dorsal horn the difference approached borderline significance (*P* = 0.07, *n* = 6). A significant increase in GFRα1-IR was also detected in the area of the SPN. No effect of injury on GFRα1-IR was detected in lumbar spinal cord (Table 1; Figures 2C,D,M), and no change was detected at either spinal level after unilateral hypogastric nerve transection (Table 1; Figures 2E–H,N). Consistent with the increase in GFRα1-IR measured quantitatively in the SPN, we observed that staining in this region was also intense and appeared to occupy a larger area in sections taken from rats that underwent joint bilateral pelvic and hypogastric nerve transections (Figures 2I–L). No further differences were identified by visual inspection of these sections.

The above observations suggested that a major effect of unilateral pelvic nerve transection was to increase GFRα1-IR in the SPN, a region that contains autonomic preganglionic neurons that project in the pelvic nerve (Hancock and Peveto, 1979; Morgan et al., 1981; Nadelhaft and Booth, 1984). We made further qualitative observations relating to this result by viewing the rostro-caudal extent of the SPN in horizontal spinal cord sections. Seven days after unilateral pelvic nerve transection GFRα1-IR fibers, and neuronal cell bodies in the SPN, were more intensely stained on the ipsilateral side (Figures 3A–C). The somata were distributed throughout the rostro-caudal extent of the SPN and were commonly observed in small clusters of up to 4–6 neurons (Figures 3F,G). More neurons were counted on the side ipsilateral to the injury (total count/section from 5 sections per rat: *ipsilateral*: 74.1 ± 5.3 and *contralateral*: 5.1 ± 1.8; *P* = 0.0002, *n* = 5). This asymmetric distribution of GFRα1-IR was not observed after bilateral nerve transection (Figures 3D,E), which resulted in more intense staining on both sides of the spinal cord.

### GFRα2 Immunoreactivity

GFRα2-IR fibers in the spinal cord contralateral to nerve injury were primarily distributed in lamina II (inner) of the dorsal horn (sacral: Figures 4A,E; lumbar: Figures 4C,G) as previously described (Kalous et al., 2007; Forrest and Keast, 2008; Keast et al., 2010). Weakly stained fibers also projected in the DGC in occasional sections but were not found in the LCP or SPN, and no GFRα2-IR neuronal cell bodies were identified.

Optical density measurements of GFRα2-IR in the sacral spinal cord 7 days after unilateral pelvic nerve transection showed no effect of injury in the lateral or medial dorsal horn (Table 1, Figures 4A,B,M). However, in the lumbar spinal cord a small but significant reduction in GFRα2-IR was detected in the medial but not the lateral dorsal horn (Table 1, Figures 4C,D,M). No change in GFRα2-IR was detected at either spinal level after unilateral hypogastric nerve transection (Table 1, Figures 4E–H,N). No differences were identified by visual inspection in sacral or lumbar spinal cord after bilateral nerve transection (Figures 4I–L).

### CGRP Immunoreactivity

CGRP-IR fibers in the spinal cord contralateral to nerve injury (sacral: Figures 5A,E; lumbar; Figures 5C,G) were primarily confined to lamina I and II and deeper laminae in the dorsal horn as previously described (Carlton et al., 1988; Lawson et al., 1993; Kalous et al., 2009). In sacral spinal cord, CGRP-IR fibers in the superficial laminae extended along the medial border of the dorsal horn and in some sections these fibers formed a continuous band following the edge of gray matter to the medial side of the opposite dorsal horn. Many CGRP-IR fibers were also present in the DGC (not shown), LCP and associated with the SPN (Figures 5A,E). In lumbar spinal cord, CGRP-IR fibers also extended along the medial border of the dorsal horn but were not as prevalent as those in sacral levels. At this spinal level, CGRP-IR fibers were not associated with the IML. At both spinal levels, CGRP-IR motor neurons were present in the ventral horn (not shown). Neuronal CGRP-IR somata were not observed in any other region.

Optical density measurements of CGRP-IR in the sacral dorsal horn 7 days after unilateral pelvic nerve transection identified a significant but small decrease in the lateral but not medial superficial dorsal horn (Table 1, Figures 5A,B,M). No effect of unilateral pelvic nerve injury was detected in the SPN (Table 1, Figure 5M) or in lumbar spinal cord (Table 1, Figures 5C,D,M). Quantification of CGRP immunostaining in sacral and lumbar spinal cord following unilateral hypogastric nerve transection showed no effect of injury on CGRP-IR at either spinal level (Table 1, Figures 5E–H,N). Following combined bilateral pelvic and hypogastric nerve transections, no further differences were identified by visual inspection in the distribution of CGRP-IR at either spinal level (Figures 5I–L).

### GFRα3 Immunoreactivity

GFRα3-IR in spinal cord dorsal horn is almost exclusively co-localized in the terminals of a major subpopulation of CGRP-IR peptidergic afferent neurons (Orozco et al., 2001; Keast et al., 2010). GFRα3-IR fibers in the spinal cord contralateral to nerve injury (sacral: Figures 6A,E; lumbar: Figures 6C,G) were distributed primarily in lamina I of the dorsal horn, consistent with previous studies (Forrest and Keast, 2008; Kalous et al., 2009; Keast et al., 2010). In sacral spinal cord, GFRα3-IR fibers projected in LCP and DGC and terminal arborizations were present in the SPN. In some sections, GFRα3-IR fibers in the superficial laminae extended along the medial border of the dorsal horn and formed a continuous band that extended to the opposite dorsal horn. GFRα3-IR fibers were not observed in the LSN. In lumbar spinal cord, GFRα3-IR fibers had a more restricted distribution and were primarily localized to lamina I (Figures 6C,G). No GFRα3-IR neuronal cell bodies were observed in either level of the spinal cord.

**Figure 7:**
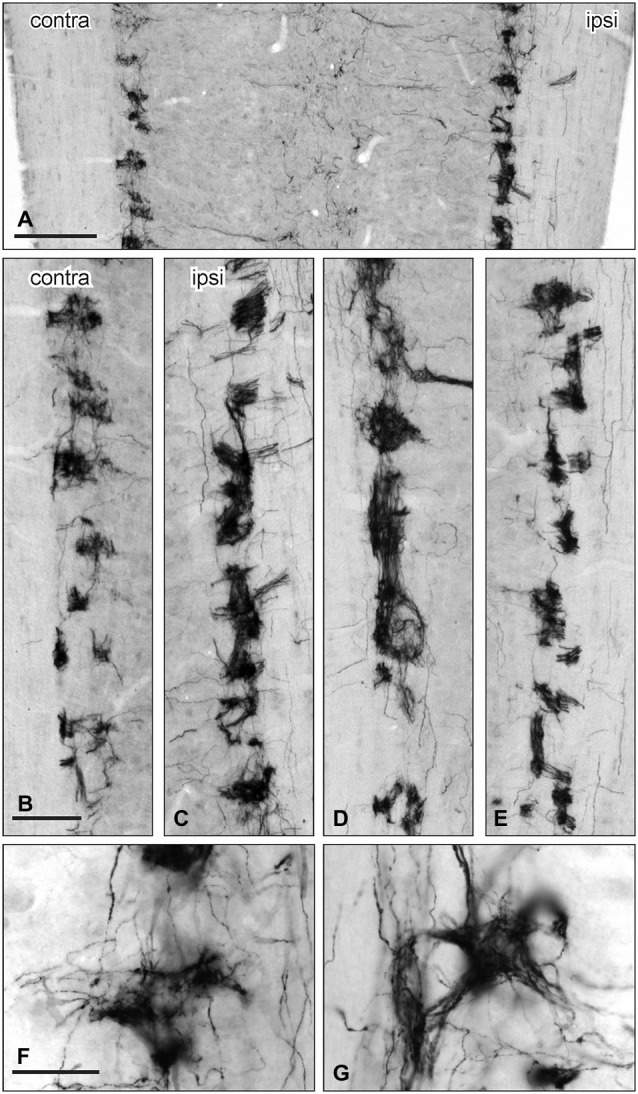
**Distribution of GFRα3-IR in the sacral parasympathetic nucleus (SPN) viewed in horizontal sections following pelvic nerve transection. (A–C)** Asymmetric upregulation of GFRα3-IR in the SPN ipsilateral to a unilateral pelvic nerve transection . **(D,E)**: Symmetric upregulation GFRα3-IR in the SPN on both sides following bilateral pelvic nerve transection. **(F,G)** GFRα3-IR axons formed dense clusters in the SPN but labeling was not see in somata. Scale bars represent 200 μm **(A)**, 50 μm **(B–E)** and 50 μm **(F,G)**.

Optical density measurements of GFRα3-IR in the sacral spinal cord 7 days after unilateral pelvic nerve transection showed no effect of injury in the lateral or medial superficial dorsal horn but did detect a significant increase in the area of the SPN (Table 1, Figures 6A,B,M, 7A–C). No effect of injury was detected in lumbar spinal cord (Table 1, Figures 6C,D,M). Quantitation of GFRα3-IR in the sacral and lumbar superficial dorsal horn 7 days after unilateral hypogastric nerve transection showed no effect of injury at either spinal level (Table 1, Figures 6E–H,N). Consistent with the increase in GFRα3-IR measured quantitatively in the SPN, we also observed that staining in this region was also intense in sections taken from rats that underwent joint bilateral pelvic and hypogastric nerve transections (Figures 6I–L) and closely resembled the side ipsilateral to unilateral pelvic nerve transection.

GFRα3-IR in the SPN was also examined in horizontal sections taken from rats that underwent unilateral pelvic or bilateral nerve transections. GFRα3-IR fibers were more intensely stained on the side ipsilateral to unilateral pelvic nerve transection but there were no immunoreactive neuronal cell bodies (Figures 7B,C). GFRα3-IR fibers also appeared more intensely stained in the SPN after bilateral pelvic nerve transection (Figures 7D,E), but this was not quantified. GFRα3-IR fibers in the SPN formed tight nests and bundles of fibers (Figures 7F,G).

## Discussion

The primary aim of this study was to determine how injuries to two major visceral nerves, the pelvic and hypogastric nerves, affect the central terminals of specific populations of sensory axons in sacral or upper lumbar spinal cord. In rat, these spinal levels are responsible for sensory and autonomic regulation of pelvic organs. We have previously found that damaging peripheral somatic sensory fibers by transecting the sciatic nerve causes extensive remodeling of their central axons in the dorsal horn of the rat spinal cord. This was detected immunohistochemically by increases in the markers GFRα1 or GFRα3 and decreases in GFRα2 but no change detected in CGRP (Keast et al., 2010). Using the same markers, this study has found the effects of injuring visceral nerves are less extensive. GFRα1-IR, a marker for a class of the non-peptidergic type of C-fiber afferent and also expressed in some myelinated afferents, was increased in the medial dorsal horn by pelvic nerve injury, and there was a small reduction in CGRP-IR, a marker of peptidergic C-fiber afferents, in the lateral dorsal horn. It is possible that in comparison to the sciatic C-fiber afferent input to lumbar dorsal horn the contribution made by pelvic and hypogastric nerves is smaller, making it more difficult to detect post-injury changes. However, we would expect this to be at least partly offset by the far more extensive projections of central visceral C-fiber axons within the spinal cord (Sugiura et al., 1989, 1993). Furthermore, we have previously reported that widespread upregulation of GFRα1-IR can be detected in sacral dorsal horn following experimental cystitis (Forrest and Keast, 2008), which should only affect a more restricted range of visceral afferents than those damaged by pelvic and hypogastric nerve transection. Our results suggest that there could be differences in how the central axons of somatic and visceral C-fiber sensory neurons respond to peripheral axotomy. Understanding these differences could provide useful understanding of biological mechanisms in somatic nociceptors that produce pathophysiology such as persistent post-surgical or neuropathic pain.

The effects of peripheral nerve injury on central terminals of C-fiber sensory neurons have been studied previously using labeling with isolectin B4-conjugates as a general marker of non-peptidergic projections (Bailey and Ribeiro-da-Silva, 2006; Casals-Díaz et al., 2009) and CGRP-IR as a marker of peptidergic projections. However, in comparison to these general markers, our group has found that using GFRα1-, GFRα2- and GFRα3-IR to identify subpopulations of each peptidergic and nonpeptidergic class provides greater anatomical resolution that reveals further specialization in the laminar projections from each population. This has also shown how each class responds independently and differently in rodent injury and inflammation models (Kalous et al., 2007, 2009; Forrest and Keast, 2008; Keast et al., 2010). However, it is important to note that our previous analyses of the lumbar (L4-L5) and lower thoracic (T9–T12) cord were aided by prior detailed reporting of the somatotopy of the sensory projections at these levels. By comparison, there is an incomplete understanding of somatotopy of the sensory input from the pelvic and hypogastric nerves in rat. HRP tracing has been used to identify sensory projections within transverse sections but not the rostrocaudal distribution of this projection across spinal cord segments. More detailed anatomical descriptions are available for cat (Morgan et al., 1981, 1986) but we are unaware of these identifying any clear somatotopic difference of the medial vs. the lateral dorsal horn.

Our analysis of the non-peptidergic type of C-fiber afferent found that GFRα1-IR increased locally in the medial dorsal horn of the sacral spinal cord after unilateral pelvic nerve transection. This was the only effect of visceral nerve injury on non-peptidergic C-fiber axons expressing GFRα1- or GFRα2-IR in this study, although it is possible that this effect occurred in the myelinated class of GFRα1-IR axons. The localized effect of visceral nerve injury on afferent fibers expressing GFRα1 contrasts with the far more extensive increase in GFRα1-IR and localized reductions of GFRα2-IR that occur in the dorsal horn of the lumbar spinal cord following sciatic nerve injury (Keast et al., 2010). Following sciatic nerve injury, these changes are accompanied by an increase in GFRα1-IR neurons in lumbar DRG, demonstrating an upregulation of the receptor protein. These different outcomes could be significant in the context of the biological activity of the endogenous ligand, a potential source of which are DRG neurons that express GDNF mRNA (Kashiba et al., 2003) and protein (Holstege et al., 1998; Jongen et al., 1999) and release it from their central terminals. GDNF administration in spinal cord is reported to have potent analgesic activity in neuropathic pain models (Boucher et al., 2000).

Following sciatic nerve injury GFRα2-IR within lumbar cord decreases due to a reduction of primary afferents expressing this receptor (Keast et al., 2010). By contrast, we found GFRα2-IR was mostly unaffected by visceral nerve transection, except for a small reduction in GFRα2-IR in the medial dorsal horn of lumbar cord after pelvic nerve transection. This effect was unexpected as we are unaware of prior evidence for the lumbar spinal region receiving sacral afferent input. We believe the contrasting effects of sciatic and visceral nerve transection on GFRα2-IR in spinal cord could be explained by this class of C-fiber neuron having relatively limited projections to the pelvic viscera. This is suggested by our previous analyses that found very few GFRα2-IR sacral afferent neurons project to the rat bladder (Forrest and Keast, 2008; Forrest et al., 2013). The biological significance of this limited projection of GFRα2-IR sensory neurons to pelvic viscera has yet to be determined.

In this study, the only effect of visceral nerve injury on the peptidergic type of C-fiber afferents was a localized reduction in CGRP-IR in the lateral dorsal horn of sacral spinal cord after pelvic nerve transection. Around one third to half of all CGRP-IR peptidergic neurons also contain GFRα3 (Kalous et al., 2009; Forrest et al., 2013). Consistent with previous studies, GFRα3-IR fibers projected only to the more superficial of the laminae that receive input from CGRP-IR fibers overall, but these were not affected by visceral nerve transection. The location in which CGRP-IR was decreased corresponds with the projection patterns of sacral afferent axons entering the dorsal horn that travel via the pelvic nerve (Morgan et al., 1981). The localized reduction in CGRP-IR could therefore be explained by selective degeneration of CGRP-IR axons that are more susceptible to axotomy. As the axons expressing GFRα3 were not affected this could be consistent with evidence that suggest the endogenous ligand, artemin, has pro-regeneration effects on sensory axons in the spinal cord (Gardell et al., 2003; Wang et al., 2008).

Unlike the L4-L5 segments of the lumbar spinal cord, the sacral (L6-S1) and upper lumbar (L1-L2) spinal cord also contain autonomic preganglionic neurons that project in the corresponding pelvic and hypogastric nerves. These autonomic regions provide another target for C-fiber input in addition to the dorsal horn. Therefore, C-fibers expressing the GDNF receptor, GFRα1, and the artemin receptor, GFRα3, are situated in close proximity to preganglionic neurons, many of which also express GFRα1 and GFRα3. The functional significance of this relationship is not known but it suggests GDNF and artemin could influence reflex functions by modulating sensory-autonomic interactions within the spinal cord. On this basis we analyzed the effects of visceral nerve injury in these spinal cord regions.

In uninjured rats, GFRα1-IR afferents project via the LCP and terminate in the region of the SPN, where small numbers of parasympathetic preganglionic neurons are also GFRα1-IR (Forrest and Keast, 2008). Other spinal cord preganglionic neurons in the thoracic IML also express both Ret and GFRα1 mRNA (Schober et al., 1999) but the role of this signaling system in normal circuit function is not known. However, there is considerable evidence supporting a role for these receptors promoting survival of sympathetic preganglionic neurons after injury (Schober et al., 1999; Schober and Unsicker, 2001). In our study, visceral nerve injury upregulated GFRα1-IR in parasympathetic preganglionic neurons in the SPN. All parasympathetic preganglionic neurons innervating the pelvic ganglia are located in the SPN (Morgan et al., 1981) and are therefore axotomised by pelvic nerve transection. Visceral nerve injury also broadly increased GFRα1- and GFRα3-IR in the SPN, but we could not determine if this was an effect on GFRα1-IR C-fiber afferent terminals or local recurrent axons projecting from preganglionic neurons. Other studies also provide evidence of neuroplasticity in these preganglionic neurons following visceral nerve injury. For example, many preganglionic neurons in the SPN that express GFRα1 also express NOS-IR (Forrest and Keast, 2008), which is up-regulated after pelvic ganglionectomy (Vizzard et al., 1995). This contrasts with expression of the acetylcholine synthesizing enzyme, choline acetyltransferase (ChAT), which is down-regulated in preganglionic neurons that express the injury markers c-Jun and ATF-3 after pelvic nerve transection (Peddie and Keast, 2011). The functional significance of the changes we observed in GFRα1- and GFRα3-IR in the SPN has yet to be determined, but again the potential role of local sensory axons as a potential source of GDNF could be considered. This locally derived GDNF could promote regeneration of injured parasympathetic preganglionic neurons or increase neuronal excitability of pelvic visceral spinal cord pathways following injury (Boucher et al., 2000; Pezet and McMahon, 2006).

This study found no effect of hypogastric nerve transection on the C-fiber sensory input or autonomic preganglionic neurons in the upper lumbar spinal cord. The hypogastric nerve contains sympathetic preganglionic axons that project to the pelvic ganglia from neurons located in L1/L2 spinal segments (Hancock and Peveto, 1979; Morgan et al., 1981). The preganglionic neurons projecting to pelvic ganglia via the hypogastric nerve are mostly (>80%) located in the DGC and not the IML (Hancock and Peveto, 1979). Unlike the SPN in sacral spinal cord, the DGC receives minimal input from GFRα1-, GFRα3- or CGRP-IR C-fiber afferents. There was not any upregulation of these markers following axotomy of lumbar afferent and sympathetic preganglionic axons in the hypogastric nerve.

## Conflict of Interest Statement

The authors declare that the research was conducted in the absence of any commercial or financial relationships that could be construed as a potential conflict of interest.

## Footnotes

^1^antibodyregistry.org

## Abbreviations

AOI: Area of interest
CGRP: Calcitonin gene-related peptide
DGC: Dorsal gray commissure
DRG: Dorsal root ganglion/ganglia
GDNF: Glial cell line-derived neurotrophic factor
GFR*α*: GDNF family receptor alpha
LCP: Lateral collateral pathway
MCP: Medial collateral pathway
SPN: Sacral parasympathetic nucleus.

## References

[B1] Airaksinen M. S. Saarma M. (2002). The GDNF family: signalling, biological functions and therapeutic value. Nat. Rev. Neurosci. 3, 383–394. 10.1038/nrn812 11988777

[B47] Anand P. Ghatei M. A. Christofides N. D. Blank M. A. McGregor G. P. Morrison J. F.. (1991). Differential neuropeptide expression after visceral and somatic nerve injury in the cat and rat. Neurosci. Lett. 128, 57–60. 10.1016/0304-3940(91)90759-m 1717899

[B2] Bailey A. L. Ribeiro-da-Silva A. (2006). Transient loss of terminals from non-peptidergic nociceptive fibers in the substantia gelatinosa of spinal cord following chronic constriction injury of the sciatic nerve. Neuroscience 138, 675–690. 10.1016/j.neuroscience.2005.11.051 16413131

[B3] Boucher T. J. Okuse K. Bennett D. L. Munson J. B. Wood J. N. Mcmahon S. B. (2000). Potent analgesic effects of GDNF in neuropathic pain states. Science 290, 124–127. 10.1126/science.290.5489.124 11021795

[B4] Carlton S. M. Mcneill D. L. Chung K. Coggeshall R. E. (1988). Organization of calcitonin gene-related peptide-immunoreactive terminals in the primate dorsal horn. J. Comp. Neurol. 276, 527–536. 10.1002/cne.902760407 3264296

[B5] Casals-Díaz L. Vivó M. Navarro X. (2009). Nociceptive responses and spinal plastic changes of afferent c-fibers in three neuropathic pain models induced by sciatic nerve injury in the rat. Exp. Neurol. 217, 84–95. 10.1016/j.expneurol.2009.01.014 19416675

[B6] Christie K. J. Zochodne D. (2013). Peripheral axon regrowth: new molecular approaches. Neuroscience 240, 310–324. 10.1016/j.neuroscience.2013.02.059 23500101

[B7] Costigan M. Scholz J. Woolf C. J. (2009). Neuropathic pain: a maladaptive response of the nervous system to damage. Annu. Rev. Neurosci. 32, 1–32. 10.1146/annurev.neuro.051508.135531 19400724 PMC2768555

[B8] Ernsberger U. (2008). The role of gdnf family ligand signalling in the differentiation of sympathetic and dorsal root ganglion neurons. Cell Tissue Res. 333, 353–371. 10.1007/s00441-008-0634-4 18629541 PMC2516536

[B9] Forrest S. L. Keast J. R. (2008). Expression of receptors for glial cell line-derived neurotrophic factor family ligands in sacral spinal cord reveals separate targets of pelvic afferent fibres. J. Comp. Neurol. 506, 989–1002. 10.1002/cne.21535 18085594 PMC3049865

[B10] Forrest S. L. Osborne P. B. Keast J. R. (2013). Characterization of bladder sensory neurons in the context of myelination, receptors for pain modulators and acute responses to bladder inflammation. Front. Neurosci. 7:206. 10.3389/fnins.2013.00206 24223534 PMC3819567

[B46] Forrest S. L. Osborne P. B. Keast J. R. (2014). Characterization of axons expressing the artemin receptor in the female rat urinary bladder: a comparison with other major neuronal populations. J. Comp. Neurol. 522, 3900–3927. 10.1002/cne.23648 25043933 PMC4167975

[B11] Gardell L. R. Wang R. Ehrenfels C. Ossipov M. H. Rossomando A. J. Miller S.. (2003). Multiple actions of systemic artemin in experimental neuropathy. Nat. Med. 9, 1383–1389. 10.1038/nm944 14528299

[B12] Hamlin A. S. McNally G. P. Osborne P. B. (2007). Induction of c-fos and zif268 in the nociceptive amygdala parallel abstinence hyperalgesia in rats briefly exposed to morphine. Neuropharmacology 343, 330–344. 10.1016/j.neuropharm.2007.05.017 17631915

[B13] Hancock M. B. Peveto C. A. (1979). Preganglionic neurons in the sacral spinal cord of the rat: an hrp study. Neurosci. Lett. 11, 1–5. 10.1016/0304-3940(79)90046-6 86176

[B14] Holstege J. C. Jongen J. L. Kennis J. H. van Rooyen-Boot A. A. Vecht C. J. (1998). Immunocytochemical localization of gdnf in primary afferents of the lumbar dorsal horn. Neuroreport 9, 2893–2897. 10.1097/00001756-199808240-00039 9760141

[B15] Jensen T. Baron R. Haanpää M. Kalso E. Loeser J. D. Rice A. S. C.. (2011). A new definition of neuropathic pain. Pain 152, 2204–2205. 10.1016/j.pain.2011.06.017 21764514

[B16] Johansen A. Romundstad L. Nielsen C. S. Schirmer H. Stubhaug A. (2012). Persistent postsurgical pain in a general population: prevalence and predictors in the tromso study. Pain 153, 1390–1396. 10.1016/j.pain.2012.02.018 22445291

[B17] Jongen J. L. Dalm E. Vecht C. J. Holstege J. C. (1999). Depletion of gdnf from primary afferents in adult rat dorsal horn following peripheral axotomy. Neuroreport 10, 867–871. 10.1097/00001756-199903170-00036 10208562

[B18] Kalous A. Keast J. R. (2010). Conditioning lesions enhance growth state only in sensory neurons lacking calcitonin gene-related peptide and isolectin b4-binding. Neuroscience 166, 107–121. 10.1016/j.neuroscience.2009.12.019 20006678

[B19] Kalous A. Osborne P. B. Keast J. R. (2007). Acute and chronic changes in dorsal horn innervation by primary afferents and descending supraspinal pathways after spinal cord injury. J. Comp. Neurol. 504, 238–253. 10.1002/cne.21412 17640046

[B20] Kalous A. Osborne P. B. Keast J. R. (2009). Spinal cord compression injury in adult rats initiates changes in dorsal horn remodeling that may correlate with development of neuropathic pain. J. Comp. Neurol. 513, 668–684. 10.1002/cne.21986 19235905

[B21] Kashiba H. Uchida Y. Senba E. (2003). Distribution and colocalization of ngf and gdnf family ligand receptor mrnas in dorsal root and nodose ganglion neurons of adult rats. Brain Res. Mol. Brain Res. 110, 52–62. 10.1016/s0169-328x(02)00584-3 12573533

[B22] Keast J. R. (2006). Plasticity of pelvic autonomic ganglia and urogenital innervation. Int. Rev. Cytol. 248, 141–208. 10.1016/s0074-7696(06)48003-7 16487791

[B23] Keast J. R. Forrest S. L. Osborne P. B. (2010). Sciatic nerve injury in adult rats causes distinct changes in the central projections of sensory neurons expressing different glial cell line-derived neurotrophic factor family receptors. J. Comp. Neurol. 518, 3024–3045. 10.1002/cne.22378 20533358 PMC2883785

[B24] Lawson S. N. Perry M. J. Prabhakar E. Mccarthy P. W. (1993). Primary sensory neurones: neurofilament, neuropeptides and conduction velocity. Brain Res. Bull. 30, 239–243. 10.1016/0361-9230(93)90250-f 7681350

[B25] Maas C. P. Trimbos J. B. Deruiter M. C. van de Velde C. J. Kenter G. G. (2003). Nerve sparing radical hysterectomy: latest developments and historical perspective. Crit. Rev. Oncol. Hematol. 48, 271–279. 10.1016/s1040-8428(03)00122-7 14693339

[B26] Morgan C. deGroat W. C. Nadelhaft I. (1986). The spinal distribution of sympathetic preganglionic and visceral primary afferent neurons that send axons into the hypogastric nerves of the cat. J. Comp. Neurol. 243, 23–40. 10.1002/cne.902430104 3950078

[B27] Morgan C. Nadelhaft I. de Groat W. C. (1981). The distribution of visceral primary afferents from the pelvic nerve to lissauer’s tract and the spinal gray matter and its relationship to the sacral parasympathetic nucleus. J. Comp. Neurol. 201, 415–440. 10.1002/cne.902010308 7276258

[B28] Nadelhaft I. Booth A. M. (1984). The location and morphology of preganglionic neurons and the distribution of visceral afferents from the rat pelvic nerve: a horseradish peroxidase study. J. Comp. Neurol. 226, 238–245. 10.1002/cne.902260207 6736301

[B29] Nadelhaft I. McKenna K. E. (1987). Sexual dimorphism in sympathetic preganglionic neurons of the rat hypogastric nerve. J. Comp. Neurol. 256, 308–315. 10.1002/cne.902560210 3558884

[B30] Navarro X. Vivo M. Valero-Cabre A. (2007). Neural plasticity after peripheral nerve injury and regeneration. Prog. Neurobiol. 82, 163–201. 10.1016/j.pneurobio.2007.06.005 17643733

[B31] Orozco O. E. Walus L. Sah D. W. Pepinsky R. B. Sanicola M. (2001). Gfralpha3 is expressed predominantly in nociceptive sensory neurons. Eur. J. Neurosci. 13, 2177–2182. 10.1046/j.0953-816x.2001.01596.x 11422460

[B32] Peddie C. J. Keast J. R. (2011). Pelvic nerve injury causes a rapid decrease in expression of choline acetyltransferase and upregulation of c-jun and atf-3 in a distinct population of sacral preganglionic neurons. Front. Neurosci. 5:6. 10.3389/fnins.2011.00006 21283532 PMC3031092

[B33] Pezet S. McMahon S. B. (2006). Neurotrophins: mediators and modulators of pain. Annu. Rev. Neurosci. 29, 507–538. 10.1146/annurev.neuro.29.051605.112929 16776595

[B34] Scheib J. Höke A. (2013). Advances in peripheral nerve regeneration. Nat. Rev. Neurol. 9, 668–676. 10.1038/nrneurol.2013.227 24217518

[B35] Schober A. Hertel R. Arumae U. Farkas L. Jaszai J. Krieglstein K.. (1999). Glial cell line-derived neurotrophic factor rescues target-deprived sympathetic spinal cord neurons but requires transforming growth factor-beta as cofactor *in vivo*. J. Neurosci. 19, 2008–2015. 10066254 10.1523/JNEUROSCI.19-06-02008.1999 PMC6782553

[B36] Schober A. Unsicker K. (2001). Growth and neurotrophic factors regulating development and maintenance of sympathetic preganglionic neurons. Int. Rev. Cytol. 205, 37–76. 10.1016/s0074-7696(01)05002-1 11336393

[B37] Schug S. A. (2012). Persistent post-surgical pain: a view from the other side of the fence. Pain 153, 1344–1345. 10.1016/j.pain.2012.02.041 22424875

[B38] Sugiura Y. Terui N. Hosoya Y. (1989). Difference in distribution of central terminals between visceral and somatic unmyelinated (c) primary afferent fibers. J. Neurophysiol. 62, 834–840. 2809705 10.1152/jn.1989.62.4.834

[B39] Sugiura Y. Terui N. Hosoya Y. Tonosaki Y. Nishiyama K. Honda T. (1993). Quantitative analysis of central terminal projections of visceral and somatic unmyelinated (c) primary afferent fibers in the guinea pig. J. Comp. Neurol. 332, 315–325. 10.1002/cne.903320305 8331218

[B40] Vizzard M. A. Erdman S. L. de Groat W. C. (1995). Increased expression of neuronal nitric oxide synthase (nos) in visceral neurons after nerve injury. J. Neurosci. 15, 4033–4045. 7538569 10.1523/JNEUROSCI.15-05-04033.1995 PMC6578258

[B41] von Hehn C. A. Baron R. Woolf C. J. (2012). Deconstructing the neuropathic pain phenotype to reveal neural mechanisms. Neuron 73, 638–652. 10.1016/j.neuron.2012.02.008 22365541 PMC3319438

[B42] Walsh P. C. Donker P. J. (1982). Impotence following radical prostatectomy: insight into etiology and prevention. J. Urol. 128, 492–497. 7120554 10.1016/s0022-5347(17)53012-8

[B43] Wang R. King T. Ossipov M. H. Rossomando A. J. Vanderah T. W. Harvey P.. (2008). Persistent restoration of sensory function by immediate or delayed systemic artemin after dorsal root injury. Nat. Neurosci. 11, 488–496. 10.1038/nn2069 18344995 PMC3417340

[B44] Yang Q. Wu Z. Hadden J. K. Odem M. A. Zuo Y. Crook R. J.. (2014). Persistent pain after spinal cord injury is maintained by primary afferent activity. J. Neurosci. 34, 10765–10769. 10.1523/jneurosci.5316-13.2014 25100607 PMC4122805

[B45] Yoshimura N. (1999). Bladder afferent pathway and spinal cord injury: possible mechanisms inducing hyperreflexia of the urinary bladder. Prog. Neurobiol. 57, 583–606. 10.1016/s0301-0082(98)00070-7 10221783

